# A multi-perspective cost-effectiveness analysis comparing rivaroxaban with enoxaparin sodium for thromboprophylaxis after total hip and knee replacement in the German healthcare setting

**DOI:** 10.1186/1472-6963-12-192

**Published:** 2012-07-09

**Authors:** Sonja Zindel, Stephanie Stock, Dirk Müller, Björn Stollenwerk

**Affiliations:** 1 Institute of Health Economics and Clinical Epidemiology of the University of Cologne, Gleueler Straße 176-178, 50935, Cologne, Germany; 2 Helmholtz Zentrum München (GmbH), Institute of Health Economics and Health Care Management, Ingolstädter Landstraße 1, 85764, Neuherberg, Germany

**Keywords:** ‘Clinical course of venous thromboembolism’, ‘Major orthopaedic surgery’, ‘Thromboembolic prophylaxis’, ‘Cost-effectiveness analysis’

## Abstract

**Background:**

Patients undergoing major orthopaedic surgery (MOS), such as total hip (THR) or total knee replacement (TKR), are at high risk of developing venous thromboembolism (VTE). For thromboembolism prophylaxis, the oral anticoagulant rivaroxaban has recently been included in the German diagnosis related group (DRG) system. However, the cost-effectiveness of rivaroxaban is still unclear from both the German statutory health insurance (SHI) and the German hospital perspective.

**Objectives:**

To assess the cost-effectiveness of rivaroxaban from the German statutory health insurance (SHI) perspective and to analyse financial incentives from the German hospital perspective.

**Methods:**

Based on data from the RECORD trials and German cost data, a decision tree was built. The model was run for two settings (THR and TKR) and two perspectives (SHI and hospital) per setting.

**Results:**

Prophylaxis with rivaroxaban reduces VTE events (0.02 events per person treated after TKR; 0.007 after THR) compared with enoxaparin. From the SHI perspective, prophylaxis with rivaroxaban after TKR is cost saving (€27.3 saving per patient treated). However, the cost-effectiveness after THR (€17.8 cost per person) remains unclear because of stochastic uncertainty. From the hospital perspective, for given DRGs, the hospital profit will decrease through the use of rivaroxaban by €20.6 (TKR) and €31.8 (THR) per case respectively.

**Conclusions:**

Based on our findings, including rivaroxaban for reimbursement in the German DRG system seems reasonable. Yet, adequate incentives for German hospitals to use rivaroxaban are still lacking.

## Background

Patients undergoing major orthopaedic surgery (MOS), such as total hip (THR) or total knee replacement (TKR), are at high risk of developing a venous thromboembolism (VTE). MOS belongs to the type of surgery with the highest VTE incidence among cardiothoracic and vascular surgery [1-3]. Serious VTE complications such as deep vein thrombosis (DVT) and pulmonary embolism (PE) usually develop within the first 3 months after surgery. In the absence of prophylaxis after hip arthroplasty, a 44% DVT risk has been reported. This was accompanied by a 3% PE risk with an all-risk mortality of 0.7% [2].

Especially at onset, VTEs may be clinically asymptomatic, making early diagnosis difficult. Therefore, routine primary prophylaxis in patients at risk of VTE is designated as a grade 1A recommendation in international guidelines [1]. For patients undergoing elective hip or knee arthroplasty, the American College of Chest Physicians recommends low-molecular-weight heparins (LMWHs), fondaparinux or a vitamin K antagonist up to 35 days after surgery. In Germany, the Association of the Scientific Medical Societies (‘Arbeitsgemeinschaft der Wissenschaftlichen Medizinischen Fachgesellschaften’, AWMF) guidelines recommend short-term 2-week prophylaxis for patients undergoing TKR and extended 5-week prophylaxis for those undergoing THR [4].

Currently, LMWHs are often used for DVT prophylaxis in Germany, in particular subcutaneous (s.c.) application of enoxaparin sodium, an antithrombin III-dependent inhibitor of factors Xa and IIa [5-7]. Because of heparin-induced thrombocytopenia (HIT), a potentially fatal complication of enoxaparin sodium treatment [8], laboratory monitoring for thrombocytes is essential. Another prophylaxis option is rivaroxaban (BAY 59–7939). In Germany, it has been marketed since October 2008 as XARELTO® and licensed for the primary prevention of postoperative thrombotic events after THR and TKR in adult patients. Rivaroxaban is an active, direct and selective antithrombin-independent factor Xa inhibitor. It is one of the first anticoagulation variants available in oral form. Because of its pharmacodynamic profile, no dosage adjustment – independent of the patient’s age, gender, body weight, or in patients with mild renal impairment – is needed [9]. It is safe to administer only one daily dose of rivaroxaban, and no specific monitoring is required [10].

Even though there are other oral active anticoagulants available for this indication, such as dabigatran etexilate, we chose enoxaparin sodium as the comparator when determining the cost-effectiveness of rivaroxaban. This choice was made because of the widespread and common use of enoxaparin sodium in German hospitals.

The efficacy of rivaroxaban has been shown in the randomized, double-blind phase III RECORD trials 1–4 published in 2008 and 2009 [11-14], which compared rivaroxaban with enoxaparin sodium in thromboembolic VTE prophylaxis strategies after MOS. Although rivaroxaban was superior to enoxaparin in preventing VTE events in all trials, no differences were found in the occurrence of major bleeding events [15].

Reimbursement of THR and TKR cases for German hospitals is based on diagnosis related groups (DRGs) [16]. DRGs are a hospital reimbursement system that classifies diagnoses with similar resource use into the same categories. Hospitals are then paid a lump sum for each case, which is calculated by the Institute for the Hospital Remuneration System (‘Institut für das Entgeltsystem im Krankenhaus’, InEK [16,17]) based on the average real resource use of selected hospitals. Since the beginning of 2011, in addition to enoxaparin sodium, rivaroxaban resource use has been weighted in the German DRG calculation scheme for THR and TKR. This means that if all hospitals initially used enoxaparin but later on collectively switched to rivaroxaban, the amount of money associated with the corresponding DRG would change in the long run. In such a case, the additional costs of rivaroxaban would be reimbursed implicitly. However, as the prescription pattern of hospitals depends on multiple conditions including but not limited to financial incentives, it is unclear whether changes in these patterns will be reimbursed.

Meanwhile, it has not yet been assessed whether reimbursement of rivaroxaban is cost-effective from the German SHI perspective. Also, it remains unclear whether financial incentives exist for German hospitals to switch from enoxaparin sodium to rivaroxaban. As the treatment costs with rivaroxaban exceed the treatment costs with enoxaparin sodium, but no additional costs are reimbursed per case, one may assume that prescribing rivaroxaban is not attractive from the hospital perspective. However, as a result of the RECORD studies, cases of VTE events can be avoided with rivaroxaban, which reduces hospital expenditure. Given the higher efficacy of rivaroxaban compared with enoxaparin sodium, German hospitals have to weigh the savings of avoided VTEs against the additional drug costs of rivaroxaban.

To analyse the cost-effectiveness of rivaroxaban compared with enoxaparin sodium in patients undergoing THR and TKR from the SHI perspective in Germany, a decision analytic model was built. Additionally, financial incentives were evaluated for German hospitals in switching from enoxaparin sodium to rivaroxaban.

## Methods

### The model

**Figure 1:**
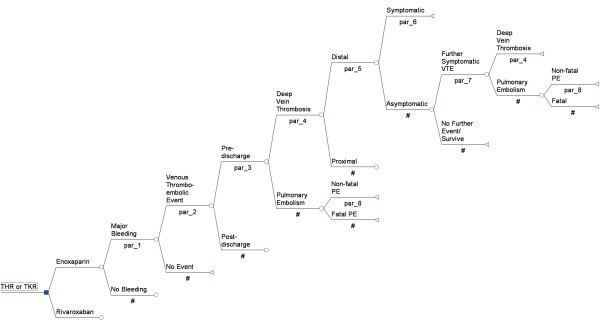
Decision analysis tree: patient outcomes during a period from surgery to 3 months postoperatively.

A decision tree was built to evaluate the cost-effectiveness of rivaroxaban versus enoxaparin sodium for thromboprophylaxis in patients undergoing THR and TKR (Figure 1). The health outcome was measured as the number of DVTs averted. All costs were measured in 2010 euros. For the analyses from the SHI perspective, we assumed that the SHI reimburses additional costs associated with the treatment of rivaroxaban. To cover the 90-day risk period for the development of postoperative VTE complications, a time horizon of 3 months after surgery was chosen [18]. In this period, all events developing during hospitalization or after discharge were included. All pathways were based on conditional probabilities reflecting the clinical course of VTE complications after MOS [2,18-23].

All patients undergoing THR and TKR are at risk of prophylaxis-related bleeding events as well as the development of a DVT or PE after surgery. All VTE events were specified by their time of incidence, whether occurring during primary hospitalization (i.e. ‘pre-discharge’) or after release from hospital (i.e. ‘post-discharge’) leading to a hospital re-admission. Irrespective of a pre- or post-discharge VTE event, patients receive outpatient follow-up treatment after hospital discharge.

According to the thrombus’s location, a distal or proximal DVT can occur. Furthermore, all DVTs were classified as asymptomatic (i.e. clinically unapparent) or symptomatic (i.e. clinically apparent) events. After prophylaxis stops, an asymptomatic DVT can change into a symptomatic VTE [20]. If a symptomatic PE is not diagnosed and treated in time, it can be fatal.

The decision tree was programmed using TreeAge Pro 2008® software (Data 3.5; TreeAge Software Inc., Williamstown, MA, USA). For statistical analyses and to assess parameter uncertainty, the software package R was used [24].

### Clinical model parameters

The probabilities of surgery-related complications such as major bleeding, distal and proximal DVT, non-fatal PE and death from any cause during the on-treatment period are based on the RECORD trials 1 and 3 [11,13]. In these multicentre, randomized, controlled, double-blind and double-dummy phase III clinical trials, rivaroxaban was compared with enoxaparin sodium in terms of effectiveness and safety in thromboprophylaxis in 12,729 patients. The primary endpoint was a composite of any DVT, non-fatal PE and death from any cause. Safety was stated in the number of prophylaxis-related major bleeding events.

The division of VTE events into ‘pre-discharge’ and ‘post-discharge’ was derived from previously published data [18]. The subdivision into symptomatic or asymptomatic DVT events as well as the VTE-dependent deaths are based on the proportions reported in the Advisory Committee Briefing Book for rivaroxaban [25]. Finally, the probability of an asymptomatic DVT becoming symptomatic was derived from previously published data [18,20].

The corresponding model parameters are summarized in Table 1.

**Table 1:** Summary of parameter distributions for probabilities used in the PSA

**Expected values of probabilities**	**Parameter**	**Expected value (standard error)**	**Source**	
		**after THR**	**after TKR**	
Prophylaxis with enoxaparin				
Major bleeding	par_1	0.002 (0.0006)	0.005 (0.0015)	[11,13]
VTE event	par_2	0.035 (0.0047)	0.187 (0.0132)	[11,13,25]
Pre-discharge VTE^1^	par_3	0.240 (0.0181)	0.530 (0.0221)	[18]
DVT	par_4	0.964 (0.0252)	0.976 (0.0120)	[11,13,25]
Distal DVT	par_5	0.415 (0.0677)	0.875 (0.0007)	[11,13,25]
Symptomatic DVT	par_6	0.167 (0.0507)	0.122 (0.0256)	[11,13,25]
Asymptomatic DVT becomes symptomatic	par_7	0.200 (0.1789)	0.048 (0.0466)	[20]
Non-fatal PE	par_8	0.500 (0.3536)	0.875 (0.1654)²	[11,13,25]
Prophylaxis with rivaroxaban				
Major bleeding	par_1	0.002 (0.0006)	0.005 (0.0015)	[11,13]
VTE event	par_9	0.010 (0.0025)	0.096 (0.0103)	[11,13,25]
Pre-discharge VTE^1^	par_3	0.240 (0.0181)	0.530 (0.0221)	[18]
DVT	par_10	0.750 (0.1083)	0.994 (0.0089)²	[11,13,25]
Distal DVT	par_11	0.917 (0.0798)	0.886 (0.0358)	[11,13,25]
Symptomatic DVT	par_12	0.250 (0.1250)	0.099 (0.0331)	[11,13,25]
Asymptomatic DVT becomes symptomatic	par_7	0.200 (0.1789)	0.048 (0.0466)	[20]
Non-fatal PE	par_13	0.875 (0.1654)²	0.500 (0.5000)²	[11,13,25]

PSA = probabilistic sensitivity analysis, VTE = venous thromboembolism, DVT = deep vein thrombosis, PE = pulmonary embolism.

^1^Values are independent of prophylactic drug.²If the point estimate yielded to 0 or 1, half an event was added or subtracted to receive reasonable distributions for probabilistic sensitivity analysis.

### Resource consumption and costs

With respect to the chosen perspectives, only direct costs are considered in our analysis. Clinical pathways for the prophylaxis, diagnosis and treatment of VTE events are based on nationally accepted AWMF guideline recommendations [4,19] and aligned with inpatient clinical protocols from available studies [26-28].

For the analysis, it was assumed that every patient receives adequate prophylaxis and, in the case of a symptomatic non-fatal VTE event, adequate diagnostic and treatment management. As 90% of patients with fatal PE die within the first 2 hours after its development [19], we assumed that these patients are not treated and incur no additional costs. Likewise, we expected asymptomatic DVTs and PEs to remain undetected and did not consider additional costs. Because there was no significant difference between major bleeding events in both the intervention and the control groups in the RECORD trials [15], the costs of major bleeding events were not considered in our analysis.

### Prophylaxis strategies

Based on current AWMF guidelines [4], prophylaxis after THR is recommended for 28–35 days and for 11–14 days after TKR. We assumed a mean prophylaxis time of 32 days and 13 days respectively. Whereas prophylaxis with s.c. enoxaparin sodium (40 mg once a day) is initiated on the day before surgery, rivaroxaban (10 mg daily) treatment starts on the day of surgery [4,11,13]. The duration of prophylaxis is limited depending on the occurrence of a bleeding complication or a VTE event. In the case of a prophylaxis-related major bleeding event during the initial treatment period, it is assumed to occur on day 5 of the hospital stay [27] and prophylaxis is stopped immediately. In the case of a pre-discharge VTE event (including DVT, non-fatal PE or fatal PE), it is assumed to occur on day 7 after surgery; prophylaxis is stopped and VTE treatment is initiated [28]. Only in the case of a post-discharge VTE event or in the absence of both a VTE event and a major bleeding event is the whole recommended prophylaxis duration (32 days after THR and 13 days after TKR) administered to the patient.

Costs of thromboembolic prophylaxis in hospital with enoxaparin sodium (i.e. drug costs, nursing time and patient education for s.c. injections, needle equipment and monitoring) were not calculated separately, as they are already included in the DRG revenues for THR and TKR [16]. Additional drug costs for rivaroxaban prophylaxis in hospital were derived from the ‘Rote Liste’, a bi-annually updated German medical drug register that provides medical professionals with summaries of drug characteristics, wholesale prices and patient information leaflets [29]. The additional drug price for rivaroxaban (€7.09) compared with enoxaparin (€4.49) was calculated as the difference in price between the drugs (€2.60) and multiplied by the days of prophylaxis duration (see Table 2).

**Table 2:** Prophylaxis duration and costs in hospital and after discharge (costs in 2010 euros)

	**Days of prophylaxis duration**	**Non-DRG included costs (€) for prophylaxis**		**Source**
		**Enoxaparin sodium**^**3**^	**Rivaroxaban**	
	**In hospital (total duration)**	**Costs for hospitals**		
MB with or without VTE^1^ - after THR- after TKR	5.0 (5)5.0 (5)	0.000.00	13.0013.00	[4,16,27,29]
Pre-discharge VTE without MB² - after THR- after TKR	7.0 (7)7.0 (7)	0.000.00	18.2018.20	[4,16,28,29]
Post-discharge VTE or no VTE and no MB - after THR- after TKR	12.4 (32)12.7 (13)	0.000.00	32.2433.02	[4,16,28,29]
	After discharge (total duration)	Costs for SHI		
Post-discharge VTE or no VTE and no MB - after THR- after TKR	19.6 (32)0.3 (13)	106.621.63	134.852.06	[4,30]

DRG = diagnosis related groups, MB = major bleeding, THR = total hip replacement, TKR = total knee replacement, VTE = venous thromboembolism, SHI = social health insurance.

^1^A major bleeding event was assumed to occur on day 5 of the hospital stay and prophylaxis is stopped immediately.

²A pre-discharge VTE event was assumed to occur on day 7 after surgery and prophylaxis is stopped.

^3^The hospital costs for enoxaparin sodium are included in the DRGs.

Costs of prophylactic drugs after hospital discharge (€6.88 for rivaroxaban and €5.44 for enoxaparin) were based on the National Association of Statutory Health Insurance Physicians (‘Kassenärztliche Bundesvereinigung’, KBV) [30] data. In conjunction with the health insurance funds, it devises and revises an office-based doctors’ fee schedule for the outpatient cost sector, the so-called German Uniform Assessment Standard (‘Einheitlicher Bewertungsmaßstab’, EbM) [30]. Drug prices per day are multiplied by the number of days the patient receives prophylaxis after discharge from hospital. Because of immediate prophylaxis withdrawal in the case of a pre-discharge VTE or major bleeding event, there is no further prophylactic drug consumption after discharge from hospital. Prophylaxis duration and associated costs are summarized in Table 2.

Additional costs of prophylaxis with rivaroxaban during hospitalization and pre-discharge VTE management are at the hospital’s expense. In contrast, costs of outpatient prophylaxis and outpatient therapy for pre-discharge VTE as well as for every post-discharge VTE event are reimbursed by the SHI.

### Diagnostics and treatment for pre-discharge VTE events

All pre-discharge VTE events including fatal PE were assumed to occur on day 7 after surgery [28]. To confirm a diagnosis of DVT, the costs of D-dimer testing and a Doppler ultrasound examination were included [19]. For diagnosis of a clinically apparent PE, blood gas analysis, D-dimer testing, chest X-ray, electrocardiogram, echocardiography and a computerized tomography (CT) scan were conducted [19].

In the case of a confirmed VTE, AWMF guidelines recommend phenprocoumon, a derivative of coumarin, for 3 months [4]. As the initial period of treatment with coumarin is associated with a procoagulant state, simultaneous LMWH treatment is administered until anticoagulation is effective [19]. In our analysis, it was assumed that patients would receive enoxaparin sodium twice a day for 6 days including thrombocyte monitoring, making further regular outpatient visits after discharge from hospital necessary. For each patient with DVT or PE, compression therapy is started in hospital and continued after discharge [4]. Prices for in-hospital diagnostic and treatment management are taken from the university teaching hospital in Cologne, Germany. Prices for medical drugs are derived from the ‘Rote Liste’ [29]. Costs of outpatient VTE follow-up therapy are based on the EbM [30].

### Additional length of stay for pre-discharge VTE events

As the incidence of a non-fatal pre-discharge VTE event requires extended hospitalization on a general ward, additional hospital days were considered. These additional hospital days (i.e. after THR: 3.9 days for DVT, 4.6 days for non-fatal PE; after TKR: 3.3 days for DVT, 6.0 days for non-fatal PE) [26] were added to the mean hospitalization time after THR (12.4 days) and TKR (12.7 days) based on the specific DRG for THR and TKR [16]. In the case of PE, it was assumed that patients would spend on average 1 day in the intensive care unit.

In the German DRG system, a hospital receives reimbursement for THR or TKR irrespective of the hospital duration between 4 and 18 days [31]. In the German healthcare system, costs for the mean hospitalization time after THR and TKR are covered by the DRG reimbursement. The hospital receives a fixed revenue irrespective of a hospital duration between 4 and 18 days [31]. Beyond this timeframe, the DRG revenue increases gradually per day. For all pre-discharge VTE complications in our analysis – except in the case of a non-fatal PE after TKR – the extended hospitalization is within this range, and costs for additional hospital days as well as further treatment management are carried by the hospitals themselves [31]. Only in the case of a non-fatal PE after TKR is an extended hospitalization time of 18.7 days in total required. In this case, the hospital receives a slightly higher DRG revenue.

Average daily hospital costs were approximated based on the average DRG reimbursement per hospital day [16]. Resource consumption and costs of pre-discharge VTE events are listed in Table 3.

**Table 3:** Resource consumption and costs (€) of pre-discharge venous thromboembolism incurred in hospital and after discharge (outpatient) (costs in 2010 euros)

**Resource consumption**		**DVT after THR (units)**	**DVT after TKR (units)**	**nf PE after THR(units)**	**nf PE after TKR(units)**	**Unit prices (€)**	**Source**
In hospital	Diagnostics						
Blood gas analysis		0.0	0.0	1.0	1.0	4.95	[19,27,28], UTHC
	D-dimer	1.0	1.0	1.0	1.0	9.90	[19,27,28], UTHC
	Doppler ultrasound	1.0	1.0	0.0	0.0	9.90	[19,27,28], UTHC
	Chest X-ray	0.0	0.0	1.0	1.0	7.70	[19,27,28], UTHC
	ECG	0.0	0.0	1.0	1.0	8.36	[19,27,28], UTHC
	CT	0.0	0.0	1.0	1.0	154.00	[19,27,28], UTHC
	Echocardiography	0.0	0.0	1.0	1.0	27.50	[19,27,28], UTHC
	Treatment						
	Enoxaparin sodium	12.0	12.0	12.0	12.0	9.98	[19,27-29]
	Subcutaneous injection	12.0	12.0	12.0	12.0	2.20	[19,27,28], UTHC
	Blood sample (TZ)	1.0	1.0	1.0	1.0	5.50	[19,27,28], UTHC
	Phenprocoumon (days)	10.3	10.0	11.0	12.7	0.17	[19,27-29]
	Anticoagulant monitoring^1^	2.0	2.0	2.0	2.0	8.80	[19,27,28], UTHC
	Compression therapy	1.0	1.0	1.0	1.0	5.23	[19,27,28], UTHC
	Additional LOS^2^ (€)	1,086.27	919.15	1,716.45	1,973.73		[4,16,19,26]
Total costs for hospital (€)		1,282.27	1,115.14	2,105.22	2,362.79		
In hospital	Extra DRG reimbursement³	0	0	0	191.46		
Outpatient	Treatment						
	Outpatient visit^4^	1.0	1.0	1.0	1.0	53.16	[19,27,28,30]
	Phenprocoumon (days)	79.7	80.0	79.0	77.3	0.17	[19,27,28,30]
	INR measurement	10.0	10.0	10.0	10.0	1.68	[19,27,28,30]
	Compression stockings	2.0	2.0	2.0	2.0	36.90	[19,27,28,30]
Total costs for health insurance (€)		157.35	157.40	157.23	348.40		

DVT = deep vein thrombosis, THR = total hip replacement, TKR = total knee replacement, nf PE = non-fatal pulmonary embolism, ECG = electrocardiogram, CT = computerized tomography with contrast agent, TZ = thrombocytes, LOS = length of stay, UTHC = University teaching hospital in Cologne, Germany.

^1^Including blood sample and INR (international normalized ratio) measurement.

²Additional hospital days are derived from Tilleul et al. [26] and calculated as 3.9 days for DVT and 4.6 days for non-fatal PE after THR;

3.3 days for DVT and 6.0 days for non-fatal PE after TKR.

³DRG reimbursement for an extended hospital stay beyond 18 days.

^4^Flat rate including all outpatient visits and blood samples every quarter (3 months).

### Diagnostics and treatment for post-discharge VTE events

We assumed that every ‘post-discharge’ DVT leads to hospital admission. In the case of a fatal PE, it was assumed that death would occur on the day of re-admission. All diagnostic and therapeutic measures including re-admissions to hospital due to ‘post-discharge’ VTE events were included in the weighted specific DRG reimbursement for DVT and PE [16]. Resource consumption and costs of post-discharge VTE events are listed in Table 4.

**Table 4:** Resource consumption and costs (€) of post-discharge VTE events after THR and TKR from the social health insurance perspective (costs in 2010 euros)

**Resources**		**DVT² (units)**	**Non-fatal PE² (units)**	**Fatal PE³(units)**	**Unit prices(€)**	**Source**
In hospital	Diagnosis, treatment and hospitalization (€)	1,838.33	3,543.48	1,256.43		[16]
Outpatient	Treatment					
	Outpatient visit^1^	1.0	1.0	0.0	53.16	[19,27,28,30]
	Phenprocoumon (days)	83.4	79.7	0.0	0.17	[19,27,28,30]
	INR measurement	10.0	10.0	0.0	1.68	[19,27,28,30]
	Compression stockings	2.0	2.0	0.0	36.90	[19,27,28,30]
Total costs for health insurance (€)		1,995.89	3,700.43	1,256.43		

VTE = venous thromboembolism, THR = total hip replacement, TKR = total knee replacement, DVT = deep vein thrombosis, PE = pulmonary embolism.

^1^Flat rate including all outpatient visits and blood samples every quarter (3 months).

²It was assumed that every post-discharge DVT and PE leads to hospital admission.

³In the case of a fatal PE, it was assumed that death would occur on the day of re-admission.

### Cost-effectiveness analysis and probabilistic sensitivity analysis

To accommodate the different settings (THR versus TKR) and perspectives (SHI versus hospital), four modifications of the model were run. The main model outcomes are defined as incremental costs and incremental effects, estimated by averaging the corresponding values resulting from probabilistic sensitivity analysis (PSA).

To reflect parameter uncertainty [32,33], a probabilistic analysis with 1,000 iterations was performed, in which all costs were assumed to be Gamma distributed and probabilities to be Beta distributed (Tables 1 and 5). Parameters of the Gamma and the Beta distributions were approximated based on the corresponding expected value and standard error. As there were no reliable stochastic estimates for the standard errors of costs, we applied 10% of the corresponding costs as the standard error for each cost parameter. The results of the PSA are displayed as a scatterplot of incremental costs and incremental effects and as cost-effectiveness acceptability curves.

**Table 5:** Summary of parameter distributions for costs (in 2010 euros) used in the PSA

**Costs of resources**	**Parameter**	**Expected costs (€) (standard error)**			
		**After THR**		**After TKR**	
		**Hospital perspective**	**SHI perspective**	**Hospital perspective**	**SHI perspective**
Prophylaxis costs with enoxaparin in the case of					
Post-discharge VTE or no VTE and no MB	par_14	0	106.42 (10.64)	0	1.63 (0.16)
Prophylaxis costs with rivaroxaban in the case of					
MB with or without VTE	par_15	13.00 (1.30)	0	13.00 (1.30)	0
Pre-discharge VTE without MB	par_16	18.20 (1.82)	0	18.20 (1.82)	0
Post-discharge VTE or no VTE and no MB	par_17	32.24 (3.22)	134.85 (13.49)	33.02 (3.30)	2.06 (0.21)
Total costs of pre-discharge VTE					
DVT	par_18	1,282.27 (128.33)	157.35 (17.74)	1,115.14 (111.51)	157.40 (15.74)
Non-fatal PE	par_19	2,105.22 (210.52)	157.23 (15.73)	2,362.79 (236.28)	348.40 (34.84)
Total costs of post-discharge VTE					
DVT	par_20	0	1,995.89 (199.59)	0	1,995.89 (199.59)
Non-fatal PE	par_21	0	3,700.43 (370.04)	0	3,700.43 (370.04)
Fatal PE	par_22	0	1,256.43 (125.64)	0	1,256.43 (125.64)

PSA = probabilistic sensitivity analysis, THR = total hip replacement, TKR = total knee replacement, SHI = social health insurance, VTE = venous thromboembolism, MB = major bleeding, DVT = deep vein thrombosis, PE = pulmonary embolism.

To estimate the impact of individual parameters on the results of the analysis, we performed an analysis of covariance (ANCOVA) [32]. Using this approach, the proportion of the sum of squares in the output parameters (i.e. incremental costs and incremental effects) explained by the variation in each input parameter was identified. Furthermore, univariate deterministic sensitivity analysis has been performed for parameters that were identified as greatly affecting the results.

## Results

**Figure 2:**
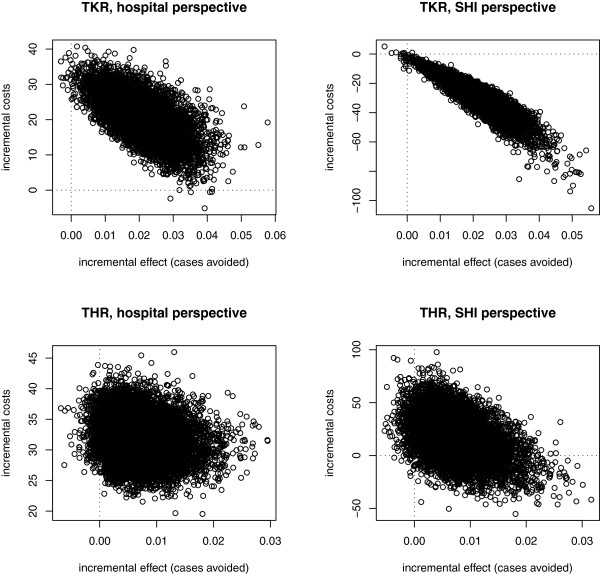
Scatterplot of incremental costs and effects.

Prophylaxis with rivaroxaban prevents on average 0.020 VTE events per person treated after TKR (95% CI: [0.007; 0.036]) and 0.007 events per person treated after THR (95% CI: [−0.0005; 0.018]). From a hospital perspective, rivaroxaban reduces the profit by €20.6 per person treated in the case of TKR (95% CI: [€9.3; €31.4]) and by €31.8 (95% CI: [€25.5; €38.7]) in the case of THR. From the SHI perspective, prophylaxis with rivaroxaban is dominant in TKR: direct costs are reduced by €27.3 (95% CI: [€9.6; €51.6]) per person treated. In THR, however, prophylaxis with rivaroxaban leads to non-significant additional costs from the SHI perspective (i.e. €17.8, 95% CI: [−€20.7; €55.6] per person treated). This results in an incremental cost-effectiveness ratio of €875 per VTE event avoided. The results after TKR are robust with respect to PSA: from the SHI perspective, 99.8% of the simulated scenarios are located in the lower right (i.e. dominant) quadrant and, from the hospital perspective, 99.8% of the simulated scenarios are located in the upper right quadrant (i.e. increasing costs for hospitals) of the cost-effectiveness plane (see Figure 2).

The scatterplots of incremental costs and effects are provided in Figure 2. Numerical estimates are provided in Tables 6 and 7. With respect to THR, there is a wider spread over the quadrants of the cost-effectiveness plane. Prophylaxis with rivaroxaban reduces the number of VTE events in 96% of the iterations of the PSA. From the SHI perspective, the costs associated with rivaroxaban are higher than the costs associated with enoxaparin in 81.6% of the cases. In 18.3% of the cases, prophylaxis with rivaroxaban was considered to be dominant over prophylaxis with enoxaparin (i.e. lower right quadrant).

**Table 6:** Results of the base case cost-effectiveness analysis (year 2010 values) from the German hospital perspective (standard errors in parentheses)

**Type of surgery**	**Costs (€)**	**Incremental costs (€)**	**VTE events per person**	**Incremental effect (events avoided)**	**ICER (€ per event avoided)**
THR					
Rivaroxaban	33.8 (3.3)	31.8 (3.4)	0.005 (0.002)	0.007 (0.005)	1,564
Enoxaparin	2.05 (0.7)		0.012 (0.005)		
TKR					
Rivaroxaban	38.8 (3.9)	20.6 (5.6)	0.014 (0.005)	0.020 (0.007)	1,014
Enoxaparin	18.1 (4.3)		0.035 (0.009)		

THR = total hip replacement, TKR = total knee replacement, VTE = venous thromboembolism, ICER = incremental cost-effectiveness ratio.

**Table 7:** Results of the base case cost-effectiveness analysis (year 2010 values) from the social health insurance perspective (standard errors in parentheses)

**Type of surgery**	**Costs (€)**	**Incremental costs (€)**	**VTE events per person**	**Incremental effect(events avoided)**	**ICER(€ per event avoided)**
THR					
Rivaroxaban	146.5 (14.2)	17.8 (19.6)	0.005 (0.002)	0.007 (0.005)	875
Enoxaparin	128.7 (14.9)		0.012 (0.005)		
TKR					
Rivaroxaban	20.7 (9.0)	−27.3 (10.7)	0.014 (0.005)	0.020 (0.007)	dominant
Enoxaparin	48.0 (16.9)		0.035 (0.009)		

THR = total hip replacement, TKR = total knee replacement, VTE = venous thromboembolism, ICER = incremental cost-effectiveness ratio.

### The cost-effectiveness acceptability curves

**Figure 3:**
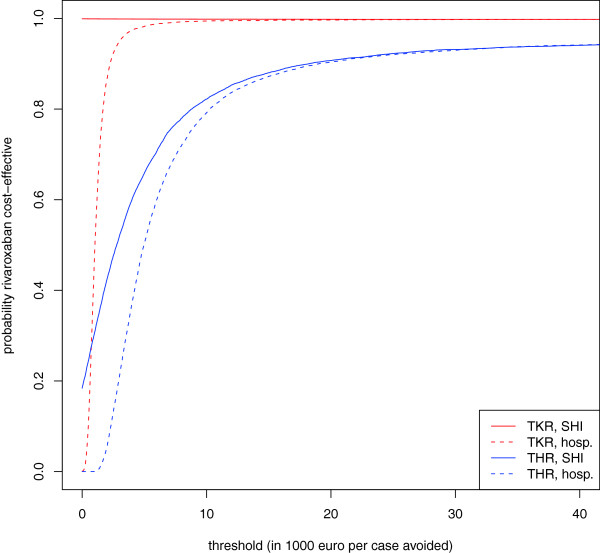
Cost-effectiveness acceptability curves.

As hospitals may not strictly minimize their treatment costs, but might balance additional treatment costs against health gain, cost-effectiveness acceptability curves are displayed for both the hospital and the SHI perspectives (Figure 3). From the hospital perspective, the probability of rivaroxaban being cost-effective differs considerably between THR and TKR. From the SHI perspective, the cost-effectiveness acceptability curves start significantly higher at a probability of 0 for a willingness-to-pay threshold of €0 per event avoided, as incremental costs are negative in 99.8% (TKR) and 18.4% (THR) of the cases (Figure 3).

### The ANCOVA analysis and deterministic sensitivity analysis

**Figure 4:**
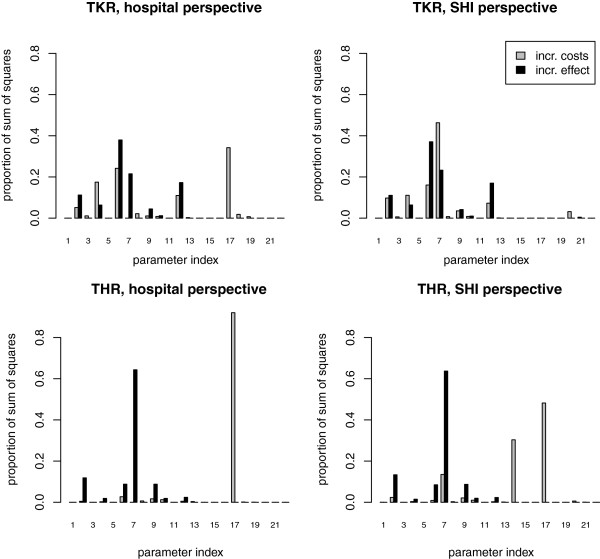
**Effect of single model parameters on the results.** Most influential parameters: Parameter 6, probability of a symptomatic deep vein thrombosis (DVT) event after prophylaxis with enoxaparin; Parameter 7, probability that an asymptomatic DVT becomes symptomatic; Parameter 17, prophylaxis costs with rivaroxaban.

Within ANCOVA analysis, several parameters were identified to explain a large proportion of the uncertainty of the results (Figure 4). Deterministic sensitivity analyses for the most influential parameters are presented in Table 8. The incremental effect (i.e. number of VTE events avoided) after TKR was mostly affected by the probability of a symptomatic DVT event after prophylaxis with enoxaparin (Parameter 6). This parameter explained 37% of the total amount of the sum of squares. The incremental effect after THR was mostly affected by the probability that an asymptomatic DVT becomes symptomatic (Parameter 7). This parameter explained 64% of the total amount of the sum of squares.

**Table 8:** Deterministic sensitivity analyses of parameters that have a large effect on the model results

	**Total hip replacement**				**Total knee replacement**			
	**Incremental events**	**Incremental costs (hospital)**	**Incremental costs (SHI)**	**ICER (SHI)**	**Incremental events**	**Incremental costs (hospital)**	**Incremental costs (SHI)**	**ICER (SHI)**
Base case	0.0070	31.8	18.0	2,569.3	0.0203	20.7	−27.2	dominance
Probability that a VTE event is a DVT during prophylaxis with enoxaparin (Parameter 4)								
0.90 0.95 0.99	0.00850.00730.0064	31.431.732.0	15.017.419.3	1,762.72,364.33,006.6	0.03220.02440.0181	6.215.723.4	−49.9−35.0−23.0	dominancedominance dominance
Probability that a DVT is symptomatic during prophylaxis with enoxaparin (Parameter 6)								
0.090.170.26	0.00490.00710.0095	32.631.830.9	21.017.914.5	4,244.12,523.91,521.3	0.01470.02860.0443	24.215.55.8	−21.8−35.3−50.4	dominance dominance dominance
Probability that an asymptomatic DVT becomes symptomatic (Parameter 7)								
0.010.150.60	0.00270.00590.0160	31.831.831.8	26.320.20.7	9,570.33,428.341.9	0.01750.02790.0613	20.720.720.7	−21.4−42.893.2	dominance dominance 1520.0
Probability that a DVT is symptomatic during prophylaxis with rivaroxaban (Parameter 12)								
0.060.160.50	0.00820.00760.0055	31.431.632.4	16.517.320.0	2,026.22,291.63,626.0	0.11230.10320.0723	18.524.143.0	−30.6−21.88.2	dominance dominance 113.3
Prophylaxis costs with enoxaparin in the case of post-discharge VTE or no VTE and no ME (Parameter 14)								
20% lower20% higher	0.00700.0070	31.8 31.8	39.2−3.2	5,592.5dominance	0.0203 0.0203	20.7 20.7	−26.9−27.5	dominance dominance
Prophylaxis costs with rivaroxaban in the case of post-discharge VTE or no VTE and no ME (Parameter 17)								
20% lower20% higher	0.00700.0070	25.438.3	−8.944.9	dominance6,402.5	0.02030.0203	14.227.2	−27.6−26.8	dominance dominance

Incremental costs were mostly affected by the prophylaxis costs with rivaroxaban (Parameter 17). These explained 92.1% of the incremental costs after THR and 34.2% after TKR from the hospital perspective. From the SHI perspective, 48.2% of the incremental costs were explained according to THR. However, with respect to TKR from the SHI perspective, the probability that an asymptomatic DVT becomes symptomatic (Parameter 7) had the highest impact on the incremental costs (46.3%).

## Discussion

Rivaroxaban was compared with enoxaparin sodium for prophylaxis of venous thromboembolism after MOS in the German healthcare setting. Based on a decision tree model, we assessed the cost-effectiveness of rivaroxaban from the SHI perspective and financial incentives from the hospital perspective.

This is the first economic evaluation of rivaroxaban in the German healthcare setting. A particular aspect of our analysis is that we simultaneously evaluated two perspectives: the German hospital perspective and the German SHI perspective. This choice was made because of the German reimbursement system: resource use of rivaroxaban was included in the German DRG calculation scheme in 2011 [16]. Despite this potential reimbursement, hospitals need incentives to change their prescription behaviour. These might not be present if the expected patient health gain is accompanied by a profit loss.

Complementing RECORD trials’ data with data from the Advisory Committee Briefing Book for rivaroxaban [25], the results of our analysis may be more precise compared with previously published analyses in which the data were based only on the RECORD trials [28,34]. Furthermore, the supplementation of probabilities, i.e. asymptomatic DVT becoming symptomatic and the differentiation of pre- and post-discharge VTE events, may offer a more detailed and comprehensive assessment of the clinical pathway of VTE events after MOS.

In our analyses, rivaroxaban was found to be more effective in the prevention of VTE events compared with enoxaparin sodium. For German hospitals, which perform 210,000 THR and 175,000 TKR each year [35], providing rivaroxaban would prevent 1,470 symptomatic VTE events in the case of THR and 3,500 events in the case of TKR. This corresponds to an annual SHI budget impact of €3.7 million (expenditure) in the case of THR and –€4.7 million (savings) in the case of TKR. With respect to the cost-effectiveness of enoxaparin from the SHI perspective, rivaroxaban was cost-saving after TKR and thus a dominant strategy. However, with respect to THR, there is still uncertainty as to whether rivaroxaban is cost-effective.

Despite these results, German hospitals are financially better off, accounting for savings through avoided VTE events, if they continue prescribing enoxaparin sodium. The cost-effectiveness acceptability curves that we presented might help hospitals in weighing image loss versus profit loss.

In terms of the effectiveness of rivaroxaban, our findings are similar to the results of other published economic models [28,34]. However, these models refer to different settings (i.e. Canada and Ireland) and use different effectiveness outcomes (i.e. quality-adjusted life–years (QALYs) and life–years gained (LYG)).

In terms of costs, the analyses conclude that prophylaxis with rivaroxaban leads to cost savings after THR and TKR from the healthcare perspective. Although this corresponds with our TKR results, we did not observe cost savings after THR. Apart from methodological reasons [36], this might be because, in Germany, medical drugs and innovations are more expensive than in many other countries.

In our analysis, the additional cost for rivaroxaban is estimated to be €2.60 per application, whereas McCullagh et al. report additional costs of €0.05 in Ireland [28], and additional costs of less than €0.50 were reported by Diamantopoulos et al. in Canada [34]. In addition, Diamantopoulos et al. calculated extra costs for the administration of enoxaparin, and they included the costs of long-term complications of VTE events in their analysis. These VTE events result in higher long-term costs of enoxaparin compared with rivaroxaban. McCullagh et al. adopted data on clinical input estimates after THR from the RECORD 2 trials, which provide a greater number of avoided VTEs with rivaroxaban. However, we did not refer to the RECORD 2 data as, in these trials, patients receive rivaroxaban prophylaxis for 35 days whereas enoxaparin was administered for only 14 days [12].

In our model, we aimed to reflect clinical treatment procedures for VTE events as realistically as possible. However, clinical pathways for prophylaxis and VTE management may differ between hospitals and limit the transferability of our results.

The model may appear to be limited in that the potential consequences of major bleeding events such as stroke or death are not considered. However, with respect to major bleeding events, no significant differences were observed between rivaroxaban and enoxaparin sodium [11,13]. By modelling the consequences of major bleeding events more explicitly, including costs and effects, they would have been cancelled out within the evaluation. However, there was still a need to include major bleeding events in the model structure. If a major bleeding event occurs, thrombosis prophylaxis stops immediately, which reduces medical expenditure for rivaroxaban or enoxaparin sodium. For model consistency, the same probability of a major bleeding event was applied for enoxaparin sodium and rivaroxaban prophylaxis. However, assuming unequal probabilities in the given tree structure did not affect the results within a sensitivity analysis (results not shown).

From the SHI perspective, the model may also appear to be limited because the potential long-term consequences of VTE such as recurrent VTE events and post-thrombotic syndrome (PTS) are not modelled. However, the incidence of long-term complications depends greatly on the aetiology of DVT. There is no evidence for the recurrence of VTE after MOS [37]. The same has been confirmed for the PTS. There is no significant difference in PTS incidence among patients with DVT or PE after THR or TKR compared with patients with no DVT history [38,39].

The use of drug prices from the ‘Rote Liste’ [29] might be viewed critically. In Germany, each hospital negotiates individual discounts with the pharmaceutical companies. As discounts can vary significantly from hospital to hospital, no representative average drug price can be estimated. In addition, hospitals do not publish their wholesale drug prices. Summarizing, it is not possible to deduce drug prices from hospital data. Hence, the drug prices given in our analysis may even lead to an overestimation of costs.

However, the bias with respect to ‘Rote Liste’ prices appears to be equal for each treatment alternative, and uncertainty is covered within PSA.

A further limitation of this evaluation is that the main effect is based on trial data. Although the study population is similar in the case of TKR (mean age 69 years (RECORD) versus 68 years (German hospitals)), in the case of THR, the patients in everyday practice are significantly older (63 years (RECORD) versus 72 years (German hospitals)) [11,13,40].

In the case of MOS, the DRG revenue includes average costs for all resource consumptions for a mean hospitalization time of 12.4 days after THR and 12.7 days after TKR. Daily costs vary from the beginning to the end of a hospital stay, as the majority of diagnostic and treatment measures are carried out within the first 2 days. However, as no precise data exist to estimate the costs of one additional hospital day, we assumed a daily cost rate based on a mean value according to the special DRG revenue. Furthermore, we did not take into account the fact that fixed costs have to be paid regardless of whether a bed is occupied or not. Finally, we did not consider the occupancy rate, which in the case of full occupancy would lead to hospital losses, as it delays the admission of further patients.

With respect to the health outcome, our analysis was conducted with the effect measure ‘number of DVTs averted’. This choice was made because the majority of German decision makers, in particular the German Institute for Quality and Efficiency in Health Care (‘Institut für Qualität und Wirtschaftlichkeit im Gesundheitswesen’, IQWiG), prefer effectiveness measures other than QALYs [41]. Furthermore, modelling QALYs would have led to a higher degree of structural uncertainty, as modelling a lifetime horizon would be substantial. In this particular case, using QALYs appeared not to be essential in drawing the final conclusions.

Furthermore, we did not consider intangible costs such as patients’ greater satisfaction with the oral administration of rivaroxaban resulting in better drug compliance [42]. As increased compliance would also decrease the number of post-operative VTE complications, our model may even underestimate cost savings with rivaroxaban prophylaxis.

Even though we recommend further research to estimate the model parameters more precisely, we believe that the uncertainty of the results is depicted accurately in our analysis. Apart from the results from the SHI perspective after THR, the results of our analysis are relatively stable, coinciding with those of other analyses [28,34].

## Conclusion

The use of rivaroxaban compared with enoxaparin reduces VTE events for both THR and TKR and results in cost savings from the SHI perspective for TKR. Based on current DRGs, profits for hospitals will decrease with the use of rivaroxaban. Considering its higher efficacy and moderate price increase compared with enoxaparin, incentives should be offered for German hospitals to prescribe rivaroxaban. Although a DRG system might give misleading incentives, these can be identified via a multiple perspective evaluation.

## Abbreviation

ANCOVA: analysis of covariance; AWMF: Association of the Scientific Medical Societies (‘Arbeitsgemeinschaft der Wissenschaftlichen Medizinischen Fachgesellschaften’); CI: Confidence interval; CT: Computerized tomography; DRG: Diagnosis related group; DVT: Deep vein thrombosis; EbM: German Uniform Assessment Standard (‘Einheitlicher Bewertungsmaßstab’); HIT: Heparin-induced thrombocytopenia; KBV: National Association of Statutory Health Insurance Physicians (‘Kassenärztliche Bundesvereinigung’); LMWHs: Low-molecular-weight heparins; LYG: Life–years gained; Mg: Milligram; MOS: Major orthopaedic surgery; PE: Pulmonary embolism; PSA: Probabilistic sensitivity analysis; QALYs: Quality-adjusted life–years; RECORD: Rosiglitazone Evaluated for Cardiac Outcomes and Regulation of Glycaemia in Diabetes; S.C.: Subcutaneous; SHI: Statutory health insurance; THR: Total hip replacement; TKR: Total knee replacement; VTE: Venous thromboembolism.

## Competing interests

The authors declare that they have no competing interests.

## Author contributions

SZ, SS and BS conceptualized and structured the decision analytic model. SZ and DM gathered the necessary data. SZ and BS ran the model. SZ, BS and SS interpreted the results. SZ and BS drafted the manuscript. All the authors read and approved the final manuscript.

## Pre-publication history

The pre-publication history for this paper can be accessed here:

http://www.biomedcentral.com/1472-6963/12/192/prepub
